# Superior temporal activation as a function of linguistic knowledge: Insights from deaf native signers who speechread

**DOI:** 10.1016/j.bandl.2009.10.004

**Published:** 2010-02

**Authors:** Cheryl M. Capek, Bencie Woll, Mairéad MacSweeney, Dafydd Waters, Philip K. McGuire, Anthony S. David, Michael J. Brammer, Ruth Campbell

**Affiliations:** a School of Psychological Sciences, University of Manchester, Manchester, UK; b Deafness, Cognition and Language Research Centre (DCAL), University College London, UK; c Institute of Cognitive Neuroscience, University College London, UK; d Institute of Psychiatry, King’s College London, UK

**Keywords:** Language processing, Semantics, Signed language, Speechreading, Deafness, Temporal cortex, Neuroimaging, fMRI

## Abstract

Studies of spoken and signed language processing reliably show involvement of the posterior superior temporal cortex. This region is also reliably activated by observation of meaningless oral and manual actions. In this study we directly compared the extent to which activation in posterior superior temporal cortex is modulated by linguistic knowledge irrespective of differences in language form. We used a novel cross-linguistic approach in two groups of volunteers who differed in their language experience. Using fMRI, we compared deaf native signers of British Sign Language (BSL), who were also proficient speechreaders of English (i.e., two languages) with hearing people who could speechread English, but knew no BSL (i.e., one language). Both groups were presented with BSL signs and silently spoken English words, and were required to respond to a signed or spoken target. The interaction of group and condition revealed activation in the superior temporal cortex, bilaterally, focused in the posterior superior temporal gyri (pSTG, BA 42/22). In hearing people, these regions were activated more by speech than by sign, but in deaf respondents they showed similar levels of activation for both language forms – suggesting that posterior superior temporal regions are highly sensitive to language knowledge irrespective of the mode of delivery of the stimulus material.

## 1 Introduction

Previous neuroimaging studies have shown that spoken language processing reliably engages the left posterior superior temporal cortex. This area comprises regions within the superior temporal plane (STP), including the planum temporale (PT; posterior to Heschl’s gyrus) and extending laterally to the posterior superior temporal gyrus and sulcus (pSTG/S) and the temporo-parieto-occipital junction (TPO) (e.g., Scott & Johnsrude, 2003). The perception of seen silent speech (speechreading) in hearing people activates superior temporal regions focused in the pSTG/S and often extending medially into the STP (Bernstein et al., 2002; Calvert, Campbell, & Brammer, 2000; Calvert et al., 1997). Similarly, in people born deaf with a signed language (SL) as their first language, SL processing elicits activation in superior temporal cortex including the PT and pSTG/S (e.g., Capek, Waters, et al., 2008; MacSweeney et al., 2002, 2004; Neville et al., 1998; Nishimura et al., 1999; Petitto et al., 2000; Sakai et al., 2005). Together, these findings suggest that the left posterior superior temporal cortex is important for meaningful language processing whether this is of visible speech or of visuo-manual sign.

In addition, this region is also involved in a number of non-linguistic processes. For example, in hearing people, non-linguistic facial actions elicit activation in the superior temporal cortex, including pSTG/S (see, for example, Pelphrey et al., 2005; Puce et al., 1998) as do other types of biological motion (for a review, see Puce & Perrett, 2003). Similarly, observation of meaningless manual gesture activates posterior superior temporal regions in observers who do not sign (Hermsdorfer et al., 2001; MacSweeney et al., 2004; Thompson et al., 2007).

While the posterior superior temporal cortex is involved in processing biological motion, activation within this region can be sensitive to the type of bodily action observed. Processing signs elicits greater activation than speech at the temporo-occipito-boundary including the posterior portions of the superior, middle and inferior temporal gyri (e.g., MacSweeney et al., 2002). These regions are also activated when non-signers observe meaningless hand actions (Hermsdorfer et al., 2001; Pelphrey et al., 2005; Thompson et al., 2007). In contrast, observing mouth movements elicits greater activation in the middle superior temporal cortex and anterior portion of the pSTG/S, for both linguistic (Capek, Waters, et al., 2008) and non-linguistic (e.g., Pelphrey et al., 2005) stimuli. One interpretation of these patterns is that cortical circuits are differentially sensitive to the visuo-articulatory correlates of the different gestural systems. While posterior parts of the lateral temporal lobe extending to the TPO prefer hand movement perception, the more anterior regions, including STP, prefer mouth movement perception (see also Thompson et al., 2007). Differential sensitivity to the articulators may also reflect variation in the amount of movement across the visual displays. There is more visual movement in sign than speech displays, and this may elicit greater activation in posterior regions involved in visual movement perception, including MT.

Thus, while a number of processes recruit the superior temporal cortex, including the perception of biological actions, the main aim of the present study was to examine the extent to which language knowledge affects cortical activation. We did this by directly contrasting patterns of activation in observers presented with displays comprising signs in BSL and spoken words. Participants either knew both BSL and spoken English, and could therefore access linguistic meaning from both types of display (deaf group) – or could speechread English but not access the linguistic content of BSL (hearing group).

Many people born deaf who acquire a SL as their first language also become proficient in a spoken language, through speechreading. By contrast, hearing people who learn a spoken language – speech monolinguals – are sensitive to the visible aspects of auditory speech (e.g., McGurk & MacDonald, 1976) and can speechread silently spoken words when these are sufficiently lexically and (visibly) phonologically distinct (Auer, 2002). However, speech monolinguals cannot access the linguistic meaning of signs. By comparing activation related to the perception of SL and of silent speech (speechreading) in both groups, we can address the extent to which exposure to a specific visible language (signs in one group, spoken words in both groups) may modify cortical activation for these inputs. While both speech and sign are likely to elicit activation in middle and posterior portions of the superior temporal cortices in both deaf and hearing participants, the relative level of activation may differ across groups and conditions. Thus, based on the neuroimaging studies described above, the following predictions were made. First, we predicted a main effect of group, with deaf participants showing greater activation than hearing participants in posterior superior temporal language regions, since deaf but not hearing participants have access to both language sources. Second, because speech is available to both groups and SL only to one, a main effect of condition was also predicted (speech greater than sign). Again, this should locate to the posterior superior temporal cortex, which previous studies have shown to be activated by both language modes in native users (e.g., MacSweeney et al., 2002). In addition, as shown previously in deaf signers (Capek, Waters, et al., 2008), speech perception should elicit greater activation than sign perception in the middle superior temporal cortex and anterior portion of the posterior superior temporal cortex. In contrast, sign perception should elicit greater activation than speech perception in the posterior portions of the superior, middle and inferior temporal cortex. Such a pattern of activation could reflect the sight of articulators.

Finally, we predict a group by condition interaction characterised by similar activation in posterior superior temporal regions to both speech and sign in the deaf group but greater activation for speech than sign in the hearing group. This prediction addresses the primary aim of our study, since it provides a strong test of our main hypothesis – that language knowledge is an important determiner of the pattern of activation in the posterior temporal cortex.

Thirteen (six females; mean age: 27.4; age range: 18–49) congenitally, severely or profoundly deaf adults were tested (81 dB mean loss or greater in the better ear over four octaves, spanning 500–4000 Hz). They were native signers, having acquired British Sign Language (BSL) from their deaf parents. Thirteen (six females; mean age: 29.4; age range: 18–43) hearing, BSL-naïve adults were also tested. All participants were right-handed with no known neurological or behavioural abnormalities.

The study formed one part of a larger experiment examining non-manual features of SL (Capek, MacSweeney, et al., 2008; Capek, Waters, et al., 2008). Participants were therefore exposed to four experimental conditions, of which two are reported here. Items were presented in the scanner in blocks of different types of material – either as silently spoken English words or as BSL signs without mouth movements. The experimental task was to respond by button press to a pre-determined target stimulus which occurred sparsely within each block. All stimuli were produced by a deaf native signer of BSL who spoke English fluently. Blocks of experimental stimuli alternated with low-level baseline comprising the model at rest. Deaf and hearing participants were given the same target-detection tasks (see Section 4).

## 2 Results

### 2.1 Behavioural results

Separate repeated-measures 2 × 2 ANOVAs for accuracy and reaction time showed that deaf native signers and hearing non-signers performed well on the target-detection task during both sign and speechreading conditions (mean correct (max = 5) [SD]): during speechreading in deaf participants = 4.69 [0.63], in hearing participants = 3.85 [0.80]; during sign in deaf participants = 5 [0], in hearing participants = 4.81 [0.25]; mean reaction time (in ms) [SD]: during speechreading in deaf participants = 1192.63 [119.22], in hearing participants = 920.08 [206.57]; during sign in deaf participants = 1252.39 [110.53], in hearing participants = 1260.07 [128.81]). Both groups were more accurate and slower at detecting targets in the sign than the speechreading condition (accuracy *F*(1, 24) = 19.331, *p* < 0.001, RT *F*(1, 24) = 33.544, *p* < 0.001). Group by condition interactions (accuracy *F*(1, 24) = 5.130, *p* = 0.033, RT *F*(1, 24) = 16.483, *p* < 0.001) showed deaf signers were more accurate at target-detection than hearing non-signers during the speechreading condition (*t*(24) = 2.994, *p* = 0.006) and also during the sign condition (*t*(24) = 2.739, *p* = 0.018). Hearing non-signers were faster than deaf signers during the speechreading condition (*t*(24) = 4.120, *p* < 0.001) but there were no group differences on the RT during the sign condition (*p* > 0.8).

### 2.2 fMRI results

**Fig. 1:**
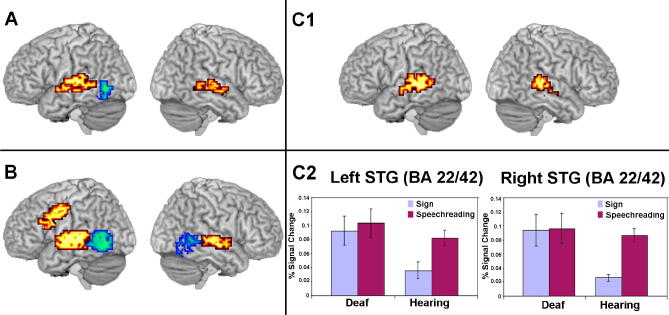
(A) Main effect of group – Deaf > Hearing (red/yellow), Hearing > Deaf (blue/green) and (B) main effect of condition – speechreading > sign (red/yellow), sign > speechreading (blue/green) and (C1) group by condition interaction (C2) Graphs display mean percent BOLD change in each condition for each group. Error bars indicate standard error of the mean (voxel-wise *p*-value = 0.025, cluster-wise *p*-value = 0.01). Activations up to 15 mm beneath the cortical surface are displayed.

The 2 × 2 ANOVA (voxel-wise *p* = 0.025; cluster-wise *p* = 0.01) showed main effects of group (deaf native signers vs. hearing non-signers) and of condition (speech vs. signs) as well as a group by condition interaction (Table 1, Fig. 1). The main effect of group showed that deaf participants displayed greater activation than hearing participants in the superior temporal cortex of both hemispheres (see Fig. 1, panel A). In the left hemisphere, the cluster of activation was focused at the border between the transverse and superior temporal gyri (BA 41/42). This cluster extended laterally into the middle and posterior portions of the superior temporal gyrus (BA 22) and inferiorly into the superior temporal sulcus and the middle temporal gyrus (BA 21). A similarly distributed cluster of activation was observed in the right hemisphere. Its focus was in superior temporal gyrus (BA 22) and it extended into BA 42 and inferiorly to the middle/posterior portion of the superior temporal sulcus and the middle temporal gyrus (BA 21).

In contrast, hearing participants displayed greater activation than deaf participants in the left posterior middle temporal gyrus (BA 37). This cluster of activation extended inferiorly to the fusiform and posterior inferior temporal gyri and posteriorly to the middle occipital gyrus (BA 19).

The main effect of condition showed that speechreading elicited greater activation than sign processing in a fronto-temporal network (see Fig. 1, panel B). In the left hemisphere, activation in the perisylvian cortex was focused at the border of the transverse and superior temporal gyri (BA 41/22). The cluster extended into posterior superior temporal cortex (BAs 22, 42) and the middle and posterior regions of the superior temporal sulcus and the middle temporal gyrus (BA 21). In the right hemisphere, activation in the temporal cortex was focused in the superior temporal gyrus (BA 22) and extended medially to include BA 42 and inferiorly to the middle and posterior portions of the superior temporal sulcus and the middle temporal gyrus (BA 21). In the left hemisphere, an additional cluster of activation was focused in the precentral gyrus (BA 6 and extended into BA 4). This cluster extended into the inferior (BAs 44, 45) and middle (BA 9) frontal gyri.

In contrast, signs elicited greater activation than speechreading in the temporo-occipital region, bilaterally. In both hemispheres, the focus of activation was in the posterior portion of the middle temporal gyrus (BA 37 and extended into BA 21). This cluster of activation extended superiorly to the superior temporal sulcus and the border of the posterior superior temporal gyrus (BA 22), inferiorly to the fusiform gyrus and posterior inferior temporal gyrus and posteriorly to the middle occipital gyrus (BA 19). In the left hemisphere, the cluster extended into the angular gyrus (BA 39). In the right hemisphere, the activation extended medially into the lingual gyrus (BA 19).

Of particular interest here is the interaction between group and condition (see Fig. 1, panel C). In hearing, but not in deaf participants, this was evident as relatively less activation for sign than for speechreading. The interaction localized to clusters of activation focused in the middle/posterior superior temporal gyri (BA 22/42), bilaterally. These clusters extended into the middle and posterior portions of the superior temporal sulcus and the middle temporal gyrus (BA 21).

In order to explore the interaction further, a secondary analysis was conducted in which the activation described in the interaction analysis was compared directly with the activation revealed by ANOVA contrasting groups for the SL condition (voxel-wise *p* = 0.05; cluster-wise *p* = 0.01). Both the interaction and follow up ANOVA showed that SL elicited greater activation in deaf than hearing participants in the posterior superior temporal gyri and sulci, bilaterally. In contrast, no regions in the interaction displayed greater activation for SL in hearing than deaf participants.

## 3 Discussion

In this study, participants viewed blocks of BSL signs and silently spoken English words in the fMRI scanner. Since both groups viewed the same stimuli and performed the same experimental tasks, differences in the patterns of activation between the groups are likely to reflect differential access to language, since deaf participants were able to process signed and silently spoken items linguistically, whereas hearing participants could only understand the spoken words.

As predicted, the main effect of *group* demonstrated greater activation in deaf than hearing participants in the middle and posterior portions of the superior and middle temporal gyri. However, factors other than language processing may contribute to this effect. In hearing people, the middle and posterior parts of the superior temporal cortex includes regions specialized for processing complex auditory information (Scott & Johnsrude, 2003). These regions can be recruited for non-linguistic visual tasks in deaf individuals (for a review, see Bavelier, Dye, & Hauser, 2006). That is, in deaf people, there may be functional plasticity in these regions resulting from the lack of auditory input. Future studies with hearing native signers will disambiguate effects of language knowledge and hearing status.

The main effect of *condition* (speechreading greater than sign) was located in a similarly distributed network including middle/posterior superior temporal regions. This may support the hypothesis that meaningful language processing, and not just deafness, shapes the functional role of superior temporal cortex in language processing, as seen speech was understood by both groups, while SL was available to just one. However, this effect may also reflect the perception of actions conveyed using different articulators – with middle/posterior superior temporal regions involved in processing oral actions and more posterior temporo-occipital regions involved in processing manual actions – irrespective of linguistic significance (see, for example, Pelphrey et al., 2005). Thus, the main effects, alone, do not permit an unequivocal conclusion that posterior superior temporal regions are involved in linguistic processing.

Seen speech also elicited greater activation than SL in the left frontal cortex including the precentral gyrus and the inferior and middle frontal gyri. This finding may reflect semantic selection or retrieval (Ruff, Blumstein, Myers, & Hutchison, 2008). Alternatively, this finding may lend support to the hypothesis that this region is involved in sub-vocal production during speech perception (for example, see Watkins, Strafella, & Paus, 2003). Moreover, Corina and colleagues (2007) showed that while deaf signers of American Sign Language (ASL) showed greater activation in the left inferior frontal gyrus for observing signs than for observing non-linguistic but meaningful actions, there was no difference in activation in this region for processing SL in deaf signers compared to hearing people who were naïve to SL (Corina et al., 2007). Future studies will test the hypothesis that the links between language perception and production may differ across language forms (speech vs. sign).

The present study also identified regions that were activated to a greater extent by SL than seen speech (Fig. 1, panel B). They included the posterior middle temporal gyrus, including V5/MT, suggesting that visual movement (or attention to visual movement; see O’Craven, Rosen, Kwong, Treisman, & Savoy, 1997) is greater for sign than for speech.

The strongest evidence in favour of the hypothesis that superior temporal regions are especially sensitive to language processing *per se* comes from the group (deaf vs. hearing) by condition (sign vs. speechreading) interaction. We predicted that an interaction showing a difference between deaf and hearing participants when viewing SL compared to viewing spoken items would indicate regions sensitive to linguistic knowledge. The superior temporal gyri displayed similar levels of activation for the perception of visible speech in both deaf and hearing groups, but different levels of activation in this region for SL. SL elicited greater activation in this region in deaf than hearing participants.

Since the current study did not include a non-linguistic biological motion condition, we cannot exclude the possibility that this difference in activation between the groups for SL (deaf > hearing) reflects the honed ability of pSTG/S to process biological motion in signers. However, the findings from previous studies have shown that SL compared to ‘pseudosign’ elicits activation in the pST cortex, bilaterally, in native signers but not in people who are naïve to SL (Neville et al., 1998; Newman et al., 2002). Thus, we argue that the greater activation in these regions for SL in deaf than hearing participants observed in the current study is unlikely due to biological motion processing alone but rather the ability of signers to process the material as semantically meaningful.

The finding that activation in the superior temporal regions was observed in both hemispheres is not surprising. Previous neuroimaging studies show that when acquired as native languages, SL (e.g., MacSweeney et al., 2002; Neville et al., 1998) and spoken language (even when presented visually) (Calvert et al., 1997; Campbell et al., 2001) processing can elicit activation in both left and right superior temporal cortices. The focus of this cluster of activation, located in the middle/posterior superior temporal gyri of both hemispheres, is spatially distinct from that of the cluster of activation we reported elsewhere (Capek, MacSweeney, et al., 2008). In that report, we showed that speechreading words elicited greater activation in deaf native signers than in hearing non-signers in the left superior temporal cortex. That cluster of activation (focused at *x* = −54, *y* = −22, *z* = 10) was located just anterior and medial to the one found in the present study (focused at *x* = −58, *y* = −30, *z* = 7). The finding that the posterior superior temporal cortex is involved in meaningful language processing irrespective of language form is consistent with models suggesting that specific linguistic processes may recruit distinctive regions within a distributed language network (Hickok & Poeppel, 2000; Scott & Johnsrude, 2003). In particular, it is probable that in order to access the meaning of a linguistic utterance, structural processes must interface with semantic ones. Posterior parts of the superior temporal cortex are highly connected with middle and inferior temporal regions that specifically support the analysis of object meaning. In addition, these regions have been implicated in lexical-semantic retrieval (see for example, Ruff et al., 2008; Vandenbulcke et al., 2007). They are also connected with both sensorimotor and articulatory regions in inferior parietal and frontal regions, thus allowing phonological representations of the utterance to be specified and maintained (see for example, Hickok & Poeppel, 2007). The extent to which distinctive subregions within posterior temporal regions are differentially involved in different aspects of *amodal* (i.e., both sign and speech) linguistic processing remains to be determined.

The posterior superior temporal cortex supports a wide range of cognitive and perceptual functions. Future research aimed at delineating functionally distinct regions within the posterior superior temporal cortex will provide insight into the precise mechanisms by which this polymodal region is involved in meaningful language processing. For now, our study suggests that this region is especially suited to processing natural language, whatever its modality.

## 4 Methods

Deaf and hearing participants were matched on non-verbal IQ as measured by the Block Design subtest of the WAIS-R (*p* > 0.1). Speechreading was tested using the Test of Adult Speechreading (TAS) (Mohammed, Campbell, MacSweeney, Barry, & Coleman, 2006), and all participants performed well, though deaf participants scored significantly higher than hearing non-signers (mean score (max = 45), deaf: 32.54, hearing: 25.08, *t*(24) = 4.779, *p* < 0.001). Despite the difference in speechreading skill, the clusters of activation identified in the interaction reported here persist even when the speechreading skill (as measured by the TAS) is entered into the analyses as a covariate. All participants gave written informed consent to participate in the study according to the Declaration of Helsinki (BMJ 1991; 302: 1194) and the study was approved by the Institute of Psychiatry/South London and Maudsley NHS Trust Research Ethics Committee.

Each condition consisted of 24 stimulus items. Between each sign, the model’s hands came to rest at his waist. The image showed a frontal view of the head, trunk and hands of the model, well lit from above, and recorded in full colour.

Stimuli were presented in alternating blocks of each of the experimental conditions, with a baseline condition lasting 30 s and 15 s respectively. The total run duration (four conditions, of which two are reported here) was 15 min. During the experimental conditions, participants were directed to pay attention to each word/sign as it occurred, and to make a push-button response whenever the stimulus item meant ‘yes’. This procedure was designed to ensure that all participants attended to every item, whether or not they were able to identify it. The ‘yes’ target was presented in an appropriate form in both conditions: as an English word with no manual component in the speechreading condition and as a BSL sign with no oral component in the sign condition (for rationale, see Capek, MacSweeney, et al., 2008). Participants were shown examples of the ‘yes’ targets outside of the scanner.

The baseline condition comprised video of the model at rest. The model’s face, trunk and hands were shown, as in the experimental conditions. Participants were directed to press a button when a grey fixation cross, digitally superimposed on the face region of the resting model, turned red. For additional stimuli and design details, see Supplementary material.

Gradient echoplanar MRI data were acquired with a 1.5-T General Electric Signa Excite (Milwaukee, WI, USA) with TwinSpeed gradients and fitted with an 8-channel quadrature head coil. Three hundred T 2∗-weighted images depicting BOLD contrast were acquired at each of the 40 near-axial 3-mm thick planes parallel to the intercommissural (AC-PC) line (0.3-mm interslice gap; TR = 3 s, TE = 40 ms). High-resolution EPI scans were acquired to facilitate registration of individual fMRI datasets to standard space. This comprised 40 near-axial 3-mm slices (0.3-mm gap), which were acquired parallel to the AC-PC line (TR = 3 s, TE = 40 ms).

The fMRI data were first corrected for motion artefact then smoothed using a Gaussian filter (FWHM 7.2 mm). In line with the non-parametric procedures used by our group, data analysis at the individual subject level used wavelet-based resampling of the time series to deal with non-independence of residuals after model fitting (see Bullmore et al., 2001). Following computation of the model fit, a goodness of fit statistic was derived by calculating the ratio between the sum of squares due to the model fit and the residual sum of squares (SSQ ratio) at each voxel. The voxel-wise SSQ ratios were calculated for each subject from the observed data and, following time series permutation, were transformed into standard space (Talairach & Tournoux, 1988) as described previously (Brammer et al., 1997; Bullmore et al., 1996).

Differences between experimental conditions were calculated by fitting the data at each voxel where all subjects had non-zero data using the following linear model: *Y = a + bX + e*, where *Y* is the vector of BOLD effect sizes for each individual, *X* is the contrast matrix for the particular inter condition/group contrasts required, *a* is the mean effect across all individuals in the various conditions/groups, *b* is the computed group/condition difference and *e* is a vector of residual errors. The model is fitted by minimising the sum of absolute deviations rather than the sums of squares to reduce outlier effects. The null distribution of *b* is computed by permuting data between conditions (assuming the null hypothesis of no effect of experimental condition) and refitting the above model. Group difference maps are computed as described above at voxel or cluster level by appropriate thresholding of the null distribution of *b*. This permutation method thus gives an exact test (for this set of data) of the probability of the value of *b* in the unpermuted data under the null hypothesis. The permutation process permits estimation of the distribution of *b* under the null hypothesis of no mean difference between groups. For additional analyses details, see Supplementary material.

## Figures and Tables

**Table 1:** 2 × 2 ANOVA for group (deaf native signers, hearing non-signers) and condition (silent speechreading, signs).

	Hemisphere	Size (voxels)	*x*, *y*, *z*	BA
*Main effect of group: Deaf > Hearing*				
Superior temporal gyrus	R	80	43, −26, 0	22
Transverse/superior temporal gyri	L	97	−54, −19, 10	41/42

*Main effect of group: Hearing > Deaf*				
Middle temporal gyrus	L	55	−43, −63, 0	37

*Main effect of condition: speechreading > signs*				
Superior temporal gyrus	R	121	51, −15, −7	22
Transverse/superior temporal gyri	L	174	−54, −15, 7	41/22
Precentral Gyrus	L	151	−47, −7, 40	6

*Main effect of condition: signs > speechreading*				
Middle temporal gyrus	L	175	−47, −63, 0	37
Middle temporal gyrus	R	154	43, −59, −3	37

*Group* × *condition*				
Superior temporal gyrus	R	75	58, −30, 10	22/42
Superior temporal gyrus	L	106	−58, −30, 7	22/42

Voxel-wise *p*-value = 0.025, cluster-wise *p*-value = 0.01. Foci correspond to the most activated voxel in each 3-D cluster.
