# Supplementary material for: Superior temporal activation as a function of linguistic knowledge: Insights from deaf native signers who speechread
Source: Brain Lang. 2010 Feb;112(2):129–34. doi: 10.1016/j.bandl.2009.10.004 (PMC3398390; doi:10.1016/j.bandl.2009.10.004)
Supplement: Supplementary material [file mmc1.doc]

**Methods**

*Stimuli and Experimental Design*

In the speechreading condition, items were selected from a series which had been established to be identifiable by hearing and deaf participants who were not scanned. Pretesting also established that all the signs were presented in citation form and were readily recognised and identified by native signers. Since the lexical norms for signed languages are scarce, and the norms for spoken English are not likely to be appropriate for native signers of BSL, the psycholinguistic parameters that pertain to the referent of the stimuli, rather than their form, were taken into account. Separate t-tests confirmed that the stimuli in the two conditions were matched on grammatical category (the majority of items were nouns or adjectives) and concreteness (p-values > 0.1).

Experimental stimuli were not repeated within the same block and presentation sequence for items was pseudorandomised. Items appeared at a rate of 15 per block, approximately one item every two seconds. To maintain vigilance, targets in both the experimental and baseline conditions occurred randomly at a rate of one per block. All participants practiced the tasks outside the scanner. During the scan, stimuli were projected onto a screen at the base of the scanner table via a Sanyo XU40 LCD projector and then projected to a mirror angled above the participant’s head in the scanner.

*Data Analysis*

Two modifications were introduced to the individual subject level data analysis described by Bullmore and colleagues (2001). First, the best fit between the weighted sum of the convolutions (computed between the observed time series at each voxel and the convolutions of two gamma variate functions (peak response at 4 and 8 sec) with the experimental design (Friston, Josephs, Rees, & Turner, 1998)) and the time series at each voxel was computed using the constrained BOLD effect model (Friman, Borga, Lundberg, & Knutsson, 2003), restricting the range of fits to those that reflect the physiological features of the BOLD response. Second, the data were permuted by the wavelet-based method described by Bullmore and colleagues (2001) with the exception that, prior to permutation, of any wavelet coefficients exceeding the calculated threshold as described by Donoho and Johnstone (1994) were removed. These were replaced by the threshold value. This step reduces the likelihood of refitting large, experimentally unrelated components of the signal following permutation.

**References**

Bullmore, E. T., Long, C., Suckling, J., Fadili, J., Calvert, G., Zelaya, F., et al. (2001). Colored noise and computational inference in neurophysiological (fMRI) time series analysis: Resampling methods in time and wavelet domains. *Human Brain Mapping, 12*(2), 61-78.

Donoho, D. L., & Johnstone, J. M. (1994). Ideal spatial adaptation by wavelet shrinkage. *Biometrika 81*(3), 425-455.

Friman, O., Borga, M., Lundberg, P., & Knutsson, H. (2003). Adaptive analysis of fMRI data. *NeuroImage, 19*(3), 837-845.

Friston, K. J., Josephs, O., Rees, G., & Turner, R. (1998). Nonlinear event-related responses in fMRI. *Magnetic Resonance in Medicine,* 39(1), 41-52.
